# MSGF-GLP: fusion method of visible and hyperspectral data for early detection of discolored standing trees

**DOI:** 10.3389/fpls.2023.1280445

**Published:** 2023-11-23

**Authors:** Hongwei Zhou, Yixuan Wu, Weiguang Wang, Jiayin Song, Guoyang Liu, Jie Shi, Hong Sun

**Affiliations:** ^1^ College of Computer and Control Engineering, Northeast Forestry University, Harbin, China; ^2^ Sichuan Jiuzhou Electric Group CO., LTD, Mianyang, Sichuan, China; ^3^ General Station of Forest and Grassland Pest Management, National Forestry and Grassland Administration, Shenyang, China

**Keywords:** discolored standing trees, data fusion methods, forest remote sensing, hyperspectral remote sensing, data processing

## Abstract

Pest and disease damage to forests cannot be underestimated, so it is essential to detect diseased trees in time and take measures to stop their spread. The detection of discoloration standing trees is one of the important means to effectively control the spread of pests and diseases. In the visible wavelength range, early infected trees do not show significant color changes, which poses a challenge for early detection and is only suitable for monitoring middle and late discolor trees. The spectral resolution of hyperspectral restricts the improvement of its spatial resolution, and there are phenomena of different spectral of the same and foreign objects in the same spectrum, which affect the detection results. In this paper, the method of hyperspectral and CCD image fusion is used to achieve high-precision detection of discoloration standing trees. This paper proposes an improved algorithm MSGF-GLP, which uses multi-scale detail boosting and MTF filter to refine high-resolution data. By combining guided filtering with hyperspectral images, the spatial detail difference is enhanced, and the injection gain is interpolated into the difference of each band, so as to obtain high-resolution and high-quality hyperspectral images. This research is based on hyperspectral and CCD data obtained from LiCHy, Chinese Academy of Forestry, Maoershan Experimental Forest Farm, Shangzhi City, Heilongjiang Province. The evaluation framework is used to compare with the other five fusion algorithms to verify the good effect of the proposed method, which can effectively preserve the canopy spectrum and improve the spatial details. The fusion results of forestry remote sensing data were analyzed using the vegetation Normalized Difference Water Index and Plant Senescence Reflectance Index. The fused results can be used to distinguish the difference between discoloration trees and healthy trees by the multispectral vegetation index. The research results can provide good technical support for the practical application of forest remote sensing data fusion, and lay the foundation for promoting the scientific, automatic and intelligent forestry control.

## 1 Introduction

Forests play a vital role in terrestrial ecosystems, not only promoting the carbon cycle but also mitigating global climate change (Li et al., 2018). As the main regulators of water, energy, and carbon cycles (Ellison et al., 2017), The forests play an indispensable role. Therefore, the problem of forest health has been widely concerned by ecologists around the world (Dash et al., 2017). Simultaneously, recent advancements in technology have facilitated the assessment of forest health (Estrada et al., 2023). Pests and diseases can cause great damage to forest ecosystems. The health of trees is infested by harmful pests, usually manifested as changes in canopy condition (Stone and Mohammed, 2017). Therefore, the state of the forest canopy is an indispensable indicator in forest health assessment systems. The trees detected with abnormal colors in the forest is an essential way to realize the detection of forest pests and diseases. Meanwhile, Monitoring the discoloring trees infected by pests and diseases is an essential means to control the spread of epidemics (Ren et al., 2022). However, the severity of its impact on pests and disease infection for forest canopy can only be determined by biological physiological sampling in the field until now, which its relies on human participation, and the time cost is high in practical applications (Hall et al., 2016). How to quickly and effectively detect forest pests and diseases in the early stages has become a key problem in forest health detection.

In recent years, with the rapid development of remote sensing satellites and air-to-ground observation technology, it can obtain multi-sensor and resolution data in the same area (Luo et al., 2022). Among them, high-resolution CCD and hyperspectral images have garnered significant attention in recent years.

Hyperspectral remote sensing images have been proposed due to the advantages of a continuous spectrum, multi-band, etc., which can obtain the spectral profile of the features while acquiring spatial data, rich spectral information, and the ability to describe the spectral characteristics of the ground cover in detail (Liao et al., 2015). The properties of ground cover can be distinguished according to different spectral characteristics. Forest diseases and pests can be detected by analyzing vegetation reflectance changes (Luo et al., 2022). However, its spatial resolution is relatively low and identification accuracy is poor. At present, hyperspectral remote sensing images have been widely used in forest vegetation type recognition (Shen and Cao, 2017), forest carbon storage estimation (Qin et al., 2021), and fire monitoring (Matheson and Dennison, 2012).

High-resolution CCD data provides more and accurate detailed texture features of forest trees as well as spatial detail information due to its higher spatial resolution, which provides higher accuracy in the identification of forest pests and diseases in the middle and late stages (Eugenio et al., 2022). The primary obstacle to utilizing this image for early-stage monitoring of forest pests and diseases lies in the scarcity of spectral information. Only trees with significant discoloration characteristics can be identified.

Through the above analysis, it can be found that the fusion scheme of high-resolution CCD and hyperspectral data can enrich the data source information, and the image obtained after fusion can have rich spectral information and high-resolution detail information, which will greatly improve the effectiveness of image data, which provide the possibility of monitoring forest pests and diseases in an early stage.

These methods have been used in the field of hyperspectral fusion (Yokoya et al., 2017). They can be mainly classified into imaging model-based, Bayesian (Wei et al., 2015), panchromatic sharpening (Loncan et al., 2015), and depth net-work-based methods. The imaging model-based methods mainly include hybrid image element decomposition as well as tensor decomposition methods. For example, The coupled non-negative matrix decomposition (CNMF) (Lanaras et al., 2015) has the advantages of clear mathematical principles and efficient program execution, but there are certain spectral distortions, and the fusion results are easily affected by matrix initialization. Bayesian fusion-based methods such as Maximum A Posteriori Probability Estimation-Stochastic Mixture Model (MAP-SMM) (Eismann and Hardie, 2005), The method is derived strictly according to mathematical theory and has the ability of prior constraints. HySure method, it gives better results in preserving edges while being able to smooth noise in homogeneous regions. The methods based on matrix factorization and Bayesian have a strong dependence on the spatial-spectral degradation model, and the degradation relationship of the spatial-spectral degradation model is not necessarily applicable to the actual situation, which affects the fusion performance of matrix factorization method and Bayesian method in practical applications, and there is spatial-spectral distortion in some practical situations. Component substitution, and multi-resolution analysis are used in panchromatic sharpening methods. Component substitution approaches are commonly used with the principal component analysis (PCA) algorithm, GS (Gram Schmidt), The advantage lies in the high-fidelity spatial details exhibited by the final fused result. However, a limitation of the component replacement method is its inability to capture local differences between images, leading to significant spectral distortions (Thomas et al., 2008). The main multi-resolution analysis (MRA) methods are Generalized Laplace Pyramid (Aiazzi et al., 2006) (GLP) and others. Multi-resolution analysis methods have the advantage of preserving spectral characteristics and can effectively solve practical problems, but there is still the problem of loss of spatial details.

Therefore, based on the GLP algorithm, this paper uses the multi-scale detail boosting to enhance the details of the high-resolution data, and combines the MTF filter to down-sample and interpolate the high-resolution data, and obtains the high-resolution detail image by processing the completed image and the detail enhanced high-resolution data. Guided filtering is used to obtain the spatial detail difference between the enhanced high-resolution image and each band, and the injection gain is generated. The corresponding difference is inserted into each band of the interpolated hyperspectral image to obtain the high-resolution detail-enhanced hyperspectral image.

The main innovative work of this aper is as follows:

(I) MSGF-GLP(Multi Scale Guided Filter - Generalized Laplace Pyramid): A new method for the fusion of high-resolution CCD and hyperspectral images is proposed, which uses a multi-scale detail boosting combined with MTF filters based on the GLP algorithm and introduces guided filtering techniques. It is verified through fusion experiments that the method has the advantage of maintaining spectral as well as spatial characteristics in image fusion.

(II) The proposed fusion of high-resolution CCD with hyperspectral images for detecting forest discolored standing trees can improve visualization/feature recognition performance and can be used for early forest pest and disease detection and health monitoring.

(III) The fused images are proposed to apply NDWI and PSRI vegetation indices to form data with enhanced information for monitoring discolored standing trees. It is a new solution for the early detection of discolored trees, and this method enhances the ability to monitor potential threats promptly and has practical applications in the early control of forest pests and diseases.

The rest of the paper is organized as follows: in Section II, the study area, image data, preprocessing steps, and an introduction to the improved algorithm are presented, and in Section III, the results of the fusion algorithm and the performance of the algorithm are analyzed. In section IV discusses that in the subsequent research, the data collected by the ground base station can be combined to detect and analyze the tree crown characteristics through multi-source data fusion, so that the collected canopy abnormal spectrum is more accurate, the abnormal spectral feature extraction is more real and reliable, and the application ability of the fusion algorithm in pest and disease, forest control and other aspects can be improved. The conclusions are presented in Section V.

## 2 Materials and methods

### 2.1 Airborne data

The data set used in this study is from the LiCHy airborne observation system of The Chinese Academy of Forestry (CAF), which has multiple data acquisition capabilities. It includes simultaneous acquisition of Hyperspectral images, LiDAR data, and CCD images.

Hyperspectral images were collected using the AISA Eagle II (Spectral Imaging Ltd., Oulu, Finland) hyperspectral sensor for the LiCHy system. It is a push broom imaging system that covers the VNIR spectral range from 400 nm to 1000 nm. A medium-format airborne digital camera system (DigiCAM-60) was selected as the CCD sensor with a spatial resolution of 0.2 m. The **Table 1** shows the equipment parameters of the adopted hyperspectral data with high resolution CCD data (Pang et al., 2016).

**Table 1:** The sensor parameters of the LiCHy system (Pang et al., 2016).

CCD: DigiCAM-60			
Frame size	8956×6708	Pixel size	6 µm
Imaging sensor size	40.30 mm×53.78 mm	Bit depth	16bits
FOV	56.2°	Focal length	50mm
Ground resolution @1000 m altitude	0.12 m		
Hyperspectral: AISA Eagle II			
Spectral range	400–970 nm	Spatial pixels	1024
Focal length	18.1 mm	Spectral resolution	3.3 nm
FOV	37.7°	IFOV	0.037°
Maximum bands	488	Frame rate	160 frames/s
Bit depth	12bits	View zenith angle range of	5-55°
		multi-angular module (MAM)	
Ground resolution (cross-track)	0.68m		
@1000 m altitude, nadir view			

**Figure 1:**
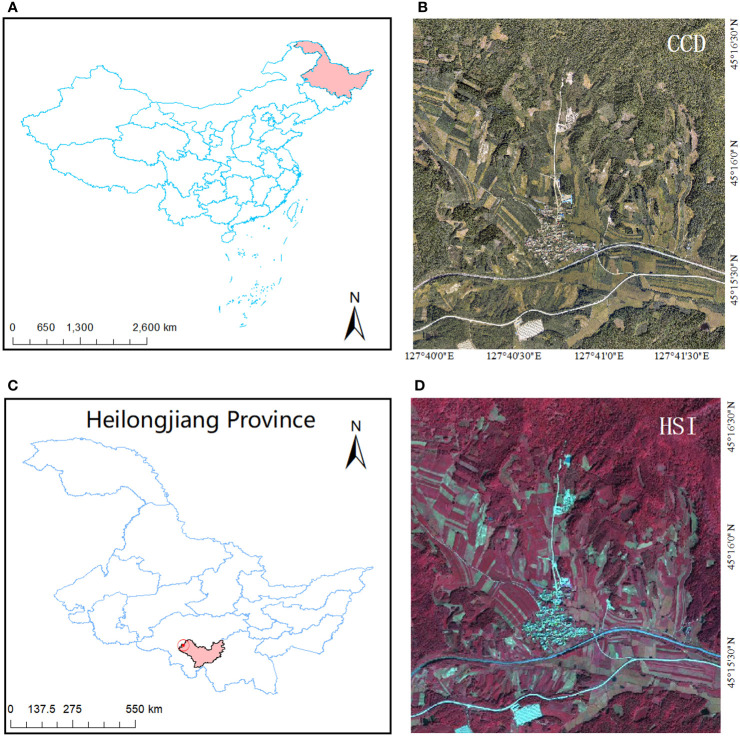
Study area location map. **(A)** Location of Heilongjiang Province in the map of China. **(B)** high-resolution CCD data of the study area. **(C)** Location of the study area in the map of Heilongjiang Province. **(D)** Hyperspectral images of the study area.

The data used in this study contained hyperspectral data, as well as CCD images. The coverage area is the Maoershan Experimental Forestry Field (127°36′E, 45°21′N) in Maoershan Town, Shangzhi City, Heilongjiang Province. The feature types are mainly man-made buildings and vegetation. The forest farm has a total area of 26,496 hectares, with an average forest coverage rate of 95% and a total forest stock of 3.5 million m³. Due to the discrete distribution of forest pests and diseases at varying degrees and diverse terrain conditions, there is a high risk of infection in this area. To effectively control the spread of forest pests and diseases and minimize losses, it is crucial to identify discolored standing trees within this forest region promptly. Therefore, the Maoershan experimental sample site has been identified as one of the most suitable areas for acquiring image data on discolored standing trees. Typical surface features such as roofs and soil exhibit high reflectance in visible and near-infrared bands while being associated with low biomass levels. Healthy green trees display higher near-infrared reflectance but lower red-band reflectance. As chlorophyll content decreases, red band reflectance increases while near-infrared reflectance decreases accordingly. These distinct spectral differences between visible-near-infrared bands enable the detection of discolored standing trees, providing a solid theoretical foundation for our study. Additionally, the selected sample site exhibits evident abnormal discoloration in wood specimens along with accurate ground features within the experimental area that facilitate precise data registration and enhance experiment accuracy. The study area is shown in **Figure 1**.

### 2.2 Image preprocessing

Given that the quality of airborne remote sensing images is influenced by various factors, including terrain conditions, flight status, and weather conditions, it becomes imperative to preprocess the acquired remote sensing data prior to data fusion. This preprocessing consists of two aspects: hyperspectral data and high-resolution CCD data.

The preprocessing of the hyperspectral data consists mainly of radiometric calibration, geometrical corrections, and atmospheric corrections. The raw AESA-Eagle hyperspectral data were judiciously resolved and calibrated using the CaligeoPRO software. Simultaneously, in combination with the calibration files of the AISA Eagle II sensor, the images were calibrated for the radiometry presented in this paper. The energy received by the sensor is not completely reflected from the ground due to atmospheric absorption and scattering of electromagnetic waves during propagation. In this paper, Using the ATCOR4 software and apply the MODTRAN model to remove atmospheric perturbations and obtain true reflectivity. High-resolution CCD data was pre-processed, including image cropping and accurate registration with hyperspectral images, to ensure the quality and effectiveness of data fusion.

### 2.3 MSGF-GLP fusion method

**Figure 2:**
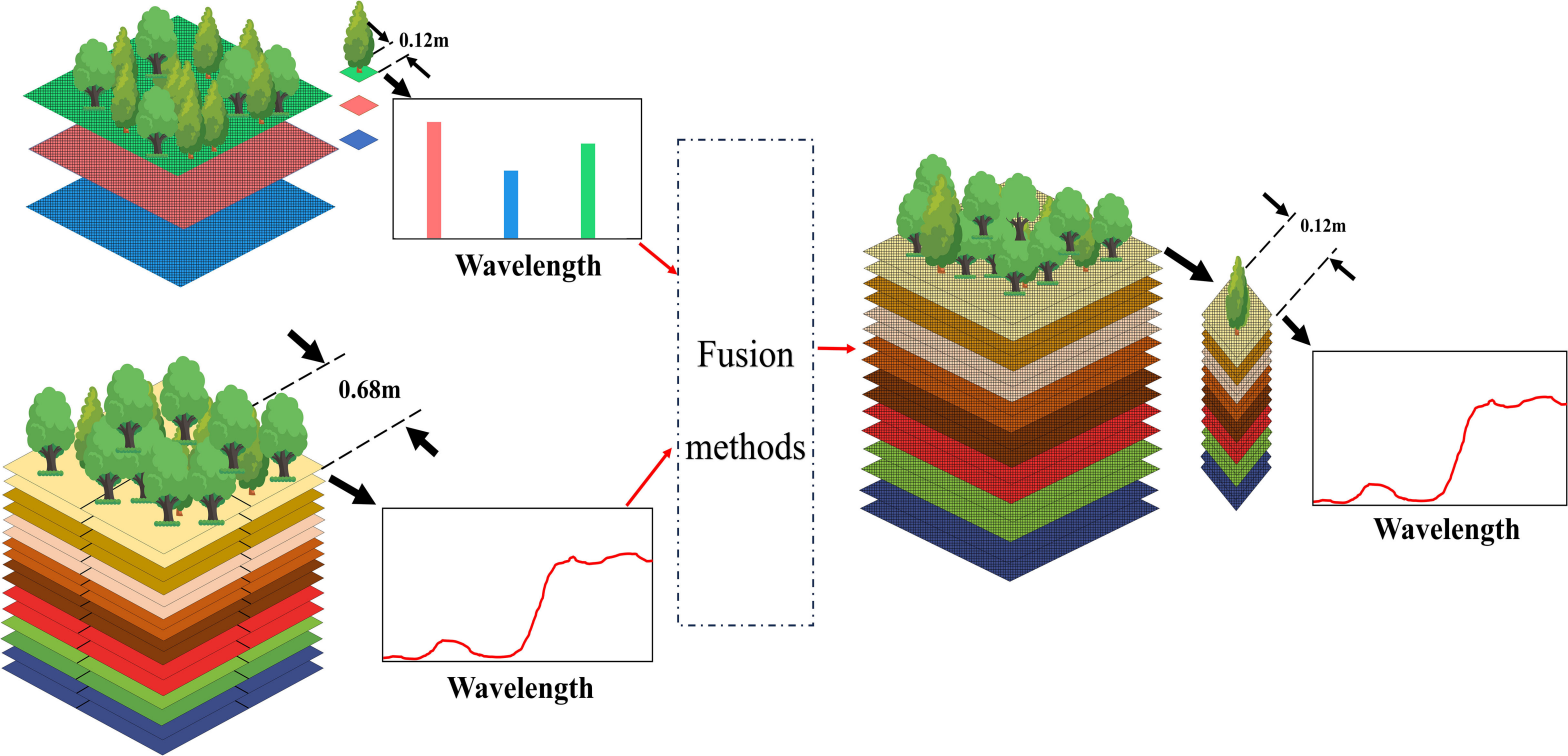
Schematic representation of the fusion result.

The fused data is designed to have both high spatial resolution as well as high spectral resolution, the effect of which is shown in **Figure 2**.

**Figure 3:**
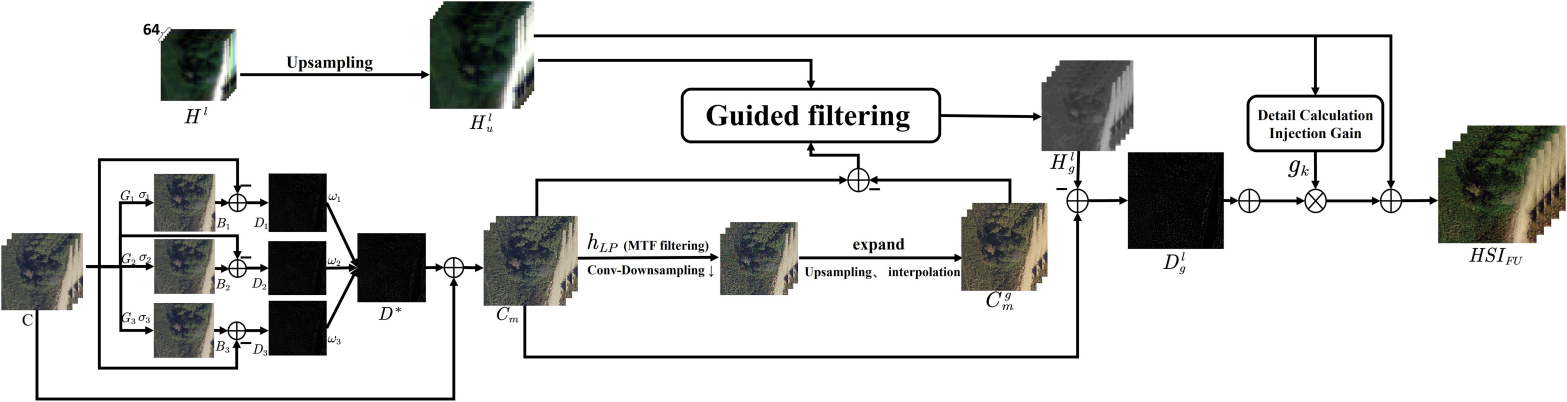
Algorithm block diagram.


Due to the indistinct details in the original image, a multi-scale detail boosting (MSDB) (Kim et al., 2015) was applied to process the high-resolution CCD data image, comprehensively enhancing its details. The processing procedure is shown in **Figure 3** as follows:

$$\{\begin{array}{c} B_{1}=G_{1}\;*\;C,\;B_{2}=G_{2}\;*\;C,\;B_{3}=G_{3}\;*\;C\\ D_{1}=C-B_{1},\;D_{2}=B_{1}-B_{2},\;D_{3}=B_{2}-B_{3}\\ D^{*}=\left(1-\omega_{1}\times \text{sgn}\left(D_{1}\right)\right)\times D_{1}+\omega_{2}\times D_{2}+\omega_{3}\times D_{3}\\ C_{m}=D^{*}+C \end{array}$$

For the original image C , three images $B_{1}$ , $B_{2}$ and $B_{3}$ with different fine scales obtained by different Gaussian filters, respectively. Where $G_{1}$ , $G_{2}$ and. $G_{3}$ are Gaussian kernel functions, and their standard deviations are taken as =1, $\sigma_{2}$ =2 and $\sigma_{3}$ =4, respectively. Then, the filtered images $B_{1}$ , $B_{2}$ and $B_{3}$ were used to generate three detailed images $D_{1}$ , $D_{2}$ and $D_{3}$ with different levels of fineness. Finally, the three detail images are merged to generate the final detail-boosting image. During this process, $\omega_{1}$ , $\omega_{2}$ , and $\omega_{3}$ $\sigma_{1}$ are chosen to be 0.5, 0.5, and 0.25, respectively, to enhance detail while suppressing saturation. Finally, the original image C is added to the overall detail image $D^{*}$ to obtain the image $C_{m}$ after detail enhancement.


$$C_{m}^{ɡ}={\text{expand}}_{r}\left(\left(C_{m}*h_{\text{LP}}\right)↓r\right)$$

Where $h_{LP}$ represents the Gaussian filter matching the hyperspectral band MTF, ↓ represents the convolution down-sampling, and the image after filtering is performed with r times of convolution down-sampling, r is the down-sampling factor, which is 8 in this paper. ${\text{expand}}_{r}$ is the interpolator, which convolutional upsample the image and interpolates it to finally obtain the image $C_{m}^{ɡ}$ .

Upsampling: The proportion of a low-resolution $H^{l}$ image that is upsampled to a CCD image. The resulting image is denoted by $H_{u}^{l}$ . Each band of the original hyperspectral image is interpolated in turn for each band of the image. Where H is the low-resolution hyperspectral image and $H_{u}^{l}$ is the l band of the interpolated hyperspectral image. $H^{l}$ is the l th band of the low-resolution image. The spectral data pixels of the original hyperspectral image pixels are transferred to the corresponding sub-pixels in the same way. Bicubic interpolation was used to upsampling the hyperspectral images.


$$H_{u}^{l}=↑H^{l}$$

Guided filter (He et al., 2013) is a kind of low-pass filter. The structure transfer property of the filter can transfer the structural details of the guided image to the input image and can eliminate the edge occlusion effect due to upsampling of the image to the maximum extent. While preserving the spectral feature information of the input image, the spatial details and texture structure of the pilot image are transferred to the output image to obtain a hyperspectral image with enhanced spatial details, which can effectively improve the quality of hyperspectral image fusion.

For the input image p , the guided image I is used as the guided filter, and the output image q is obtained after filtering. For the pixel at position i , the filtered output is a weighted average, and the guided filter is expressed by the following formula:


$$q_{i}=\sum_{j}W_{ij}\left(I\right)p_{j}$$

$W_{ij}$ is the filter kernel associated only with the guided image I.

For an input image p , the output image is q after filtering using the bootstrap image I of the bootstrap filter. The bootstrap filter assumes that the output image and the bootstrap image I satisfy a linear relationship.


$$q_{i}=a_{k}I_{i}+b_{k},\forall i\in \omega_{k}$$

where $I_{i}$ and $q_{i}$ denote the value of the i th pixel in the guide image and the output image, respectively. $\omega_{k}$ is a local window of size (2r+1) × (2r-1) with coefficients $a_{k}$ and $b_{k}$ as its linear coefficients, which are considered to be constant within $\omega_{k}$ . the radius of $\omega_{k}$ is r. Minimization of the serial port $\omega_{k}$ by the cost function.


$$E\left(a_{k},b_{k}\right)=\sum_{i\in w_{k}}\left({\left(a_{k}I_{i}+b_{k}-p_{i}\right)}^{2}+\varepsilon a_{k}^{2}\right)$$

where ϵ is an adjustable regularization parameter and $a_{k}$ and $b_{k}$ can be found by a linear regression equation.


$$\{\begin{array}{c} a_{k}=\frac{\frac{1}{\left|w\right|}\sum_{i\epsilon w_{k}}\left(I_{i}p_{i}-\mu_{i}{\bar{p}}_{k}\right)}{\sigma_{k}^{2}+\varepsilon }\\ b_{k=}{\bar{p}}_{k}-a_{k}\mu_{k} \end{array}$$

where and are the mean and variance of the corresponding bootstrap image in $w_{k}$ , respectively, is the number of all pixel points contained in $w_{k}$ , and is the mean value of image p in $w_{k}$ .

In this paper, $GF_{\varepsilon ,r}\left(\text{G},\text{P}\right)$ stands for guided filtering processing, G and P represent the guiding image and the input image respectively, and the parameter r and the orthogonalization parameter ϵ represent the radius and blur degree of the filtering window, respectively. The parameters are set to r =20 and ε =10^-6^.


$$H_{ɡ}^{l}=GF_{\varepsilon ,r}\left(H_{u}^{l},\left(C_{m}-C_{m}^{g}\right)\right)$$

$$D_{ɡ}^{l}=C_{m}-H_{ɡ}^{l}$$

Multiresolution methods obtain spatial details by decomposing high spatial resolution CCD images at multiple scales. The upsampling hyperspectral bands will be incorporated through injection. proportionally to the CCD image size. After subsampling the CCD image using the MTF filter, the interpolation calculation is performed, and then the detail image is calculated by subtracting the obtained low-resolution CCD image from the original CCD image. Guided filtering is performed on the detail image and the hyperspectral to transfer the structural details to the hyperspectral to obtain the filtered result. The hyperspectral filtered detail image is calculated by subtracting it from the original CCD image. Finally, these detailed images are added to the original hyperspectral bands to obtain high-resolution hyperspectral images.


$$HSI_{FU}^{l}=H_{u}^{l}+{ɡ}_{l}\left(C_{m}-H_{ɡ}^{l}\right)$$

$${ɡ}_{l}=\frac{H_{u}^{l}}{\frac{1}{N}\sum_{l=1}^{N}H_{u}^{l}}$$

for *l* = 1,2,…, where l HSI represents the l th band of the fused image.

### 2.4 Comparison experiment

MAP-SMM (Hardie et al., 2004) uses maximum a posteriori estimation method and random mixture model to improve the spatial resolution of hyperspectral images with the assistance of high-resolution CCD images-Sharpening Spectral. The method enhances CCD images with a high spatial resolution by sharpening them. In the experiment, hyper-spectral data was used as input for low spatial resolution, while CCD served as input for high spatial resolution. Resampling was set to cubic convolution. CNMF (Yokoya et al., 2012) estimates endmembers and high-resolution abundance maps by alternating unmixing hyperspectral and high-resolution CCD images via nonnegative matrix factorization NMF (Lee and Seung, 1999). The hyperspectral data is initialized by unmixing the hyperspectral images using VCA(Nascimento and Dias, 2005). The final high-resolution hyperspectral data are obtained by the product of spectral features and high-resolution abundance maps. HySure (Simoes et al., 2015) to formulate the fusion problem as a convex set minimization problem involving two quadratic terms and an edge-preserving term.

In the method requiring PSF, a Gaussian filter with an FWHM of GSD was used based on the FWHM provided in the data (Yokoya et al., 2017). HySure used the PSF estimation method described in(Simoes et al., 2015). The non-negative least squares method was used for estimation in the method requiring SRF (Finlayson and Hordley, 1998).

Six fusion methods are used in the experiments, including CNMF, HySure, MAP-SMM, PC-Spectral sharpening, GLP, and the improved algorithm proposed in this paper. The fusion experiment involves preprocessing high-resolution CCD data and hyperspectral data to obtain enhanced high-resolution CCD images and corrected hyperspectral data, which serve as inputs for the algorithm. Simultaneously, experimental parameters are set, and ultimately the fusion results of each algorithm are obtained. The results are evaluated using qualitative and quantitative indicators.

### 2.5 Quality evaluation

The evaluation of the effects of hyperspectral fusion images is a crucial step in fusion processing, encompassing two primary aspects: qualitative assessment and quantitative analysis (Dong et al., 2022). Qualitative evaluation needs to be combined with quantitative evaluation for a more accurate and reasonable assessment of the results of hyperspectral remote sensing fusion images.

#### 2.5.1 Qualitative evaluation

The qualitative assessment of remote sensing fusion data is conducted through direct visual inspection by the reader to discern its strengths and limitations. Visual interpretation can be used to assess the quality of fusion, but it is greatly affected by the individual knowledge of the observer, which is subjective and incomplete. However, it can provide an intuitive visual sense of the spatial resolution and sharpness of the image.

The qualitative evaluation mainly includes detecting whether there is ghost and distortion in the image, whether the fusion results are effectively preserved and enhanced in the spatial detail expression, the colour brightness and texture features of the ground objects, and whether the sharpness of the image after fusion is improved.

#### 2.5.2 Quantitative evaluation

The quantitative evaluation index of the fusion image can only represent the quality evaluation results of the fusion image in one aspect. The advantages and dis-advantages of various fusion algorithms can be found by comparing and analysing the changes in the image before and after fusion with some technical indexes. Therefore, in this study, (Spectral Angle Mapping)SAM (Alparone et al., 2007),which is used to assess the degree of spectral distortion during fusion. (Error Relative Global Adimensionnelle de Synthesse)ERGAS (Du et al., 2007),which is used to measure the global spectral quality of fused images. (Correlation Coefficient)CC (van der Meer, 2006) is reflects the degree of correlation between the fused image and the reference image. Entropy is an evaluation index of how much information the image contains. (Root Mean Square Error) RMSE is the proximity between the fused result image and the reference image, and other indicators were used to compare the images before and after fusion. The reference data are the resampled raw hyperspectral data.

#### 2.5.3 PSRI, NDWI vegetation index and canopy spectral

In addition to the above qualitative and quantitative evaluations, this paper selects PSRI, NDWI vegetation index, and tree canopy spectral curves to analyse the fused results from the spectral level. To a certain extent, they can reflect the difference between the original hyperspectral data and the fusion results and present the spectral fidelity more intuitively.

The vegetation index is defined as a dimensionless index, commonly a ratio, linear or nonlinear combination of spectral reflectance of two or more bands and is considered a sign of the relative abundance and activity of green vegetation in terms of radiance and is a comprehensive representation of chlorophyll content and green biomass of green vegetation, which is intended to diagnose the vegetation growth status and green vegetation vigour, enhances a particular attribute or characteristic of the vegetation (Munnaf et al., 2020).

The spectral reflectance changes of vegetation in the visible-NIR band after being stressed by pests and diseases are a direct feature of remote sensing of pests and diseases (Sankaran and Ehsani, 2011). Such spectral responses caused by pests and diseases are widely used in remote sensing monitoring and early stress diagnosis (Prabhakar et al., 2011). When vegetation leaves are infected, it will be accompanied by changes in chlorophyll and carotenoid content and affect the canopy water content.

Therefore, two planting indices selected in this paper: PSRI (Merzlyak et al., 1999) and NDWI (Gao, 1996), are selected to synthesize the results of fusion, as indicators to reasonably evaluate the fusion results from the spectral level as well as in practical applications.

NDWI is a normalized water index, which is used to study the water content of vegetation, and it can effectively extract the water content of the vegetation canopy and can have a more obvious response when the vegetation canopy is under water stress.


$$NDWI=\frac{GREEN-NIR}{GREEN+NIR}$$

GREEN is the green band and NIR is the near-infrared band. In this paper, the GREEN wavelength was selected as 525 nm and the NIR wavelength was 956 nm.

PSRI is the plant senescence reflectance index, which is detected using the ratio of carotenoids to chlorophyll. It can be used for vegetation health monitoring, plant physiological stress ability detection, etc.


$$PSRI=\frac{\left(\rho_{680}-\rho_{500}\right)}{\rho_{750}}$$

The canopy spectral curves can reflect the physiological properties of the features (Li et al., 2015), and comparing the canopy curves before and after fusion allows for a reasonable assessment of the actual performance of the fusion algorithm and a comparison of the changes in the actual spectra.

## 3 Results and evaluation

### 3.1 Qualitative evaluation

#### 3.1.1 Spatial detail evaluation

**Figure 4:**
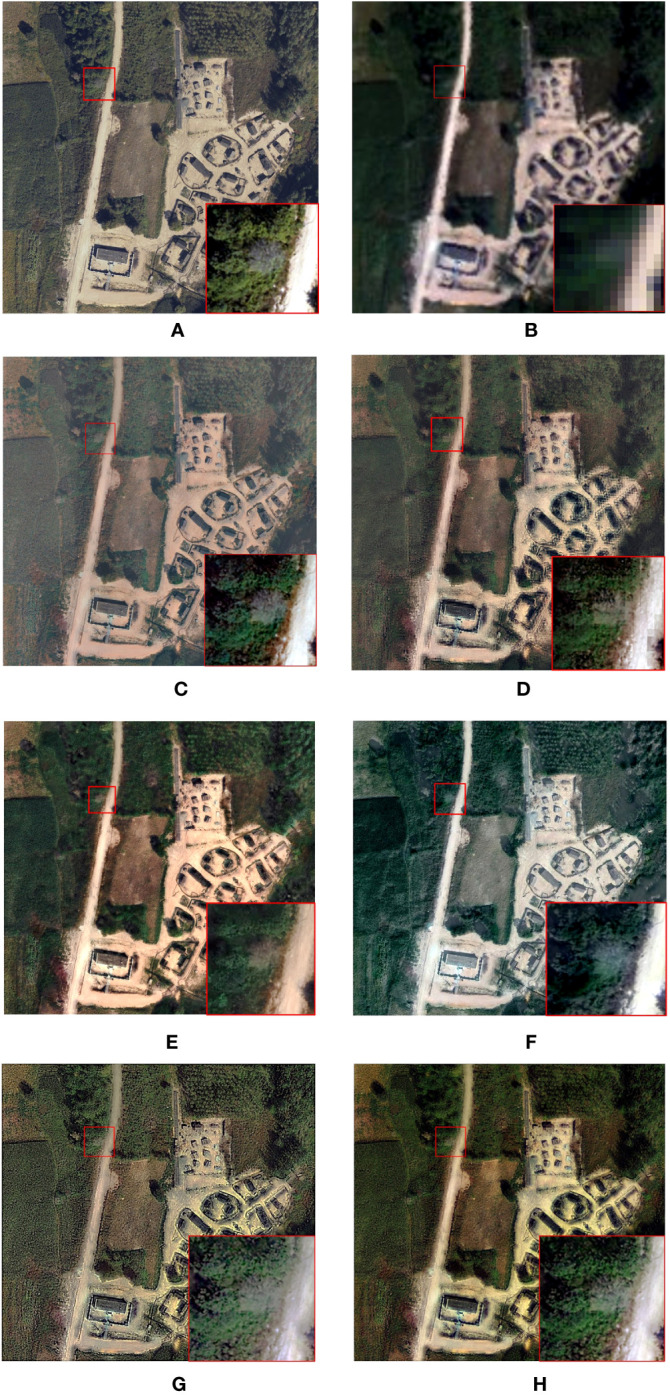
Fusion results: **(A)** CCD image **(B)** HSI data **(C)** HySure result **(D)** MAP-SMM result **(E)** CNMF result **(F)** PC Spectral sharpening result **(G)** GLP result **(H)** Proposed result.

The following is the spatial detail evaluation after the fusion is completed. As the **Figure 4** shows the original CCD image, the false colour image of the original hyperspectral data (R:666 nm; G:525 nm; B:434 nm). For the overall fusion results, there are certain colour as well as brightness differences between different fusion algorithms compared to the original data. However, the spatial texture as well as the detailed features are somewhat preserved in each fusion algorithm when compared to the local magnification.

The fusion result of HySure exhibits higher brightness than the original CCD im-age, and the feature display effect has been moderately enhanced. The fusion results from PC Spectral Sharpening are relatively fewer sharp than the initial CCD data, with lighter color tones in the vegetation parts, deviating from the other results in terms of color fidelity. Nevertheless, spatial detail information is partially preserved. The proposed algorithm preserves spatial details while enhancing the sharpness of edges in the fused image.

The MAP-SMM algorithm preserves the texture properties of tree crowns for the description of color-changing tree crowns. Although there are some differences with the original data, the HySure algorithm is able to better preserve the optical texture and features of the canopy, and the spatial features perform nicely, making the edges of the canopy and additional surrounding trees distinctly visible. In contrast, the CNMF algorithm does not perform as well in terms of detail, but can still distinguish the edge features of the discolored standing tree. The results of PC-Spectral Sharpening fusion show that the color description of the canopy of color-changing trees is not clear enough and does not effectively distinguish normal trees from their boundaries. At the same time, the spatial details are insufficient. The GLP algorithm improves the de-tailed information of tree crowns to some extent. Overall, the proposed algorithm effectively preserves and characterizes the complex and diverse details of the tree and its surroundings, and accurately distinguishes normal from abnormal conditions.

In summary, the spatial resolution of the above six algorithms is effectively im-proved compared with the original hyperspectral data, and the algorithm proposed in this paper, while improving the spatial details, can portray the abnormal canopy of discolored trees.

#### 3.1.2 Spectral level evaluation

**Figure 5:**
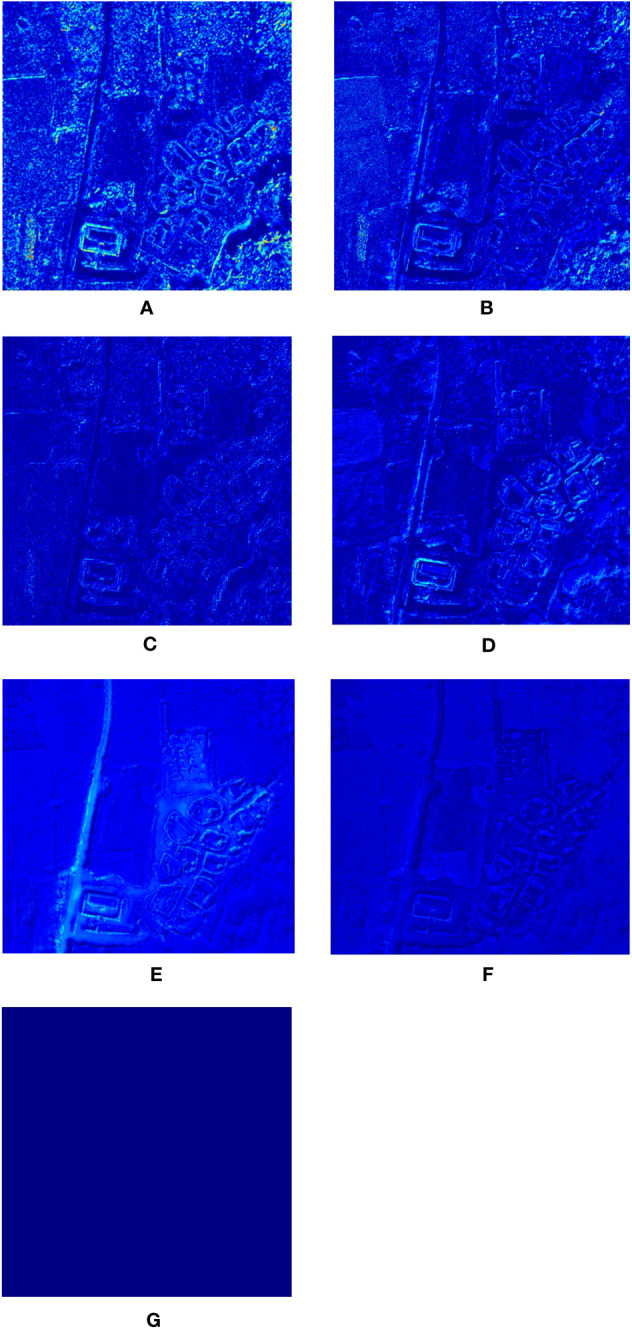
Error plot of the fusion result: **(A)**: HySure result **(B)** CNMF result **(C)**: MAP-SMM result **(D)**: PC Spectral Sharpening result **(E)**: GLP result **(F)**: Proposed result **(G)**: Reference result.

In order to be able to compare the fusion results of each method more obviously from the visual effect, the following **Figure 5** gives the difference plots between the fused hyperspectral data of each method and the reference hyperspectral with high spatial resolution in turn.

The results of the fusion error maps show that the algorithms in this paper show a low degree of spectral distortion and loss of spatial details in most of the image regions. the results of MAP-SMM are better, while CNMF, HySure, PC-Spectral Sharpening, and other algorithms show different degrees of spectral distortion and loss of spatial details. HySure can improve the spatial HySure can improve the ability of spatial de-tail expression, but there are some differences with the original data at the spectral level.

**Figure 6:**
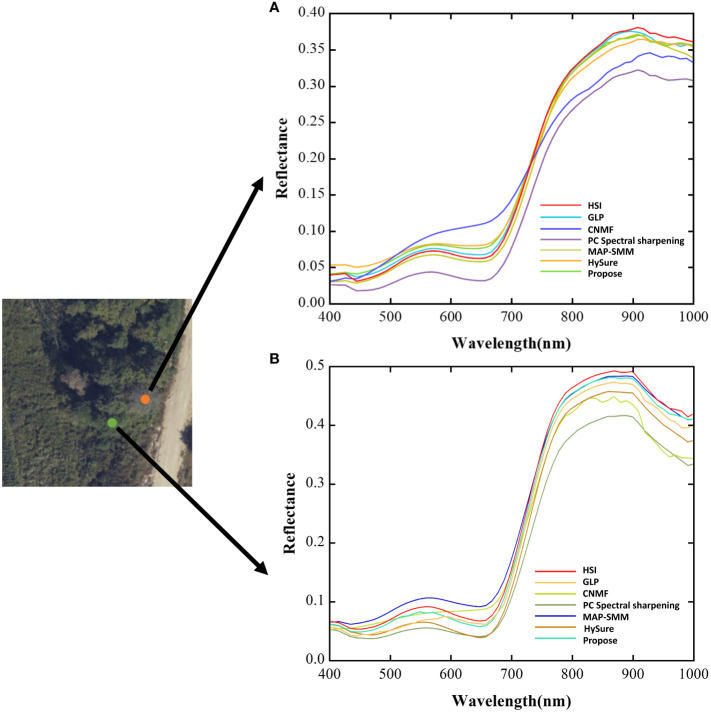
Spectral curves of trees: **(A)**: discoloured trees **(B)**: normal trees.

The following **Figure 6** shows the spectral curves of the tree canopy before and after fusion for each algorithm.

Based on the spectral curve of the fusion result, the following result can be obtained.

The fusion results of the MAP-SMM algorithm have good spectral fidelity, small differences between them and the original data, and similar trends in the spectral curves, which can preserve their valid spectral information. In the visible band, the normal tree canopy spectral curves differ slightly from the raw data. The fusion results of the HySure algorithm can have a high degree of overlap with the original hyper-spectral data in the NIR band, and the trends of the spectral curves are similar. The CNMF algorithm has some differences in the trends of its spectral curves with the original hyperspectral data in the visible band, and the spectral curves fluctuate in the NIR band. the PC Spectral Sharpening has more significant spectral differences from the original data in the spectral curves of vegetation, and there is a certain degree of spectral distortion with the same visual presentation results, but it is able to retain the trend of the spectral curves in the visible as well as near-infrared bands to some extent. The differences between the GLP algorithm and the raw data in the canopy spectral curves are smaller, the trends are similar, and there is some degree of overlap in the spectral curves.

The canopy spectral curve of this algorithm is slightly different from the original data and has a high degree of overlap with the original super-spectral data, which effectively preserves the spectral information.

The aforementioned algorithms can all enhance spatial resolution to some extent, while preserving their spectral information to varying degrees. According to the spectral curve results, the proposed algorithm can effectively preserve the canopy spectral information in terms of spectral fidelity. The overall trend of the spectra can be preserved and the difference between the pre- and post-fusion spectra is relatively small.

### 3.2 Quantitative evaluation

The purpose of hyperspectral image fusion is to combine the spatial information contained in high spatial resolution images with hyperspectral data to improve the spatial information in the final fusion result. The reference data are the raw hyperspectral data after resampling, and the strengths and weaknesses of the various fusion algorithms are evaluated and validated according to the specific metrics in **Table 2**.

**Table 2:** Fusion results.

Title 1	SAM	ERGAS	CC	RMSE	Q	Entropy
Reference	0	0	1	0	1	11.9569
CNMF	1.746	2.353	0.849	0.0721	0.8477	14.6223
PC Spectral Sharpening	6.763	3.532	0.836	0.0769	0.8268	11.9977
HySure	5.344	3.786	0.799	0.0974	0.7782	14.9207
MAP-SMM	2.852	1.548	0.922	0.5359	0.9205	14.7519
GLP	2.529	3.326	0.862	0.2485	0.7631	14.5832
Proposed	1.632	2.828	0.944	0.1495	0.8641	15.0819

The statistical metrics in the table were compared with the original hyperspectral data to quantitatively evaluate the results, and the fusion results were evaluated with quantitative metrics, including SAM, RMSE, ERGAS, CC, Entropy, and Q.

It can be seen from the table that the algorithm proposed in this paper performs well in the indicators SAM, CC, and Entropy. In general, the MAP-SMM algorithm has better performance, but it is not as good as the method proposed in this paper in the expression of spatial information. From the spectral index level analysis, the difference between CNMF and MSGF-GLP in terms of the spectral information available in the fusion result and the reference image sampled on the simulation of the original data is not significant, and the spectral characteristics of the original data are effectively preserved. The fusion results of MAP-SMM and HySure have some spectral differences compared with the reference image, but the difference is small. The difference between PC Spectral sharpening and the reference image is the largest, and the spectral distortion is more significant when combined with the qualitative evaluation result map. From the perspective of spectral loss, MAP-SMM has the least spectral loss and has a better ability to retain spectral in-formation. MSGF-GLP has less spectral distortion, which can effectively retain the spectral information in hyperspectral data and reduce the information loss in fusion. The CNMF also has less spectral distortion. The fusion results of HySure and GLP indicate some spectral loss. The PC Spectral sharpening method has more serious spectral loss than other algorithms.

Image information entropy is used to represent the increased degree of information of the fused image, and to measure the richness between the fusion result and the original image. When the information of high-resolution data is fused to hyperspectral data, the information from six algorithms is improved compared with the original data. Among them, the fusion result entropy of the algorithm proposed in this paper is the highest, indicating that the amount of information increases more, and the level of detail expression is more abundant. Followed by HySure, MAP-SMM, CNMF, and GLP, it shows that the fusion algorithm can improve the information of the original image data. The PC Spectral Sharpening algorithm improves the amount of information the least, and its spatial information expression and detail description have a certain lack compared with other algorithms. The correlation coefficient of the proposed algorithm is the largest, followed by CNMF and MAP-SMM algorithms, indicating that the fusion result can better retain its Spectral characteristics. The correlation coefficient of HySure and PC Spectral Sharpening with the original image is relatively low, which indicates that there is a certain difference between the original hyperspectral data and the HySure and PC spectral sharpening.

### 3.3 NDWI and PSRI vegetation index

The fusion results were analyzed and evaluated at the spectral level. The selected vegetation indices make the comparison of the results clearer at the spectral level.

Sapes, Gerard, et al. (Sapes et al., 2022) demonstrated that multispectral indices associated with physiological decline were able to detect differences between healthy and diseased trees. In the original high-resolution data, it was not possible to detect the wilted trees using true-color images from which their specific infected status could be determined. Therefore, this paper selects two different vegetation indices and extracts the spectral curves of the features for a comprehensive analysis and comparison of the fusion results.

**Figure 7:**
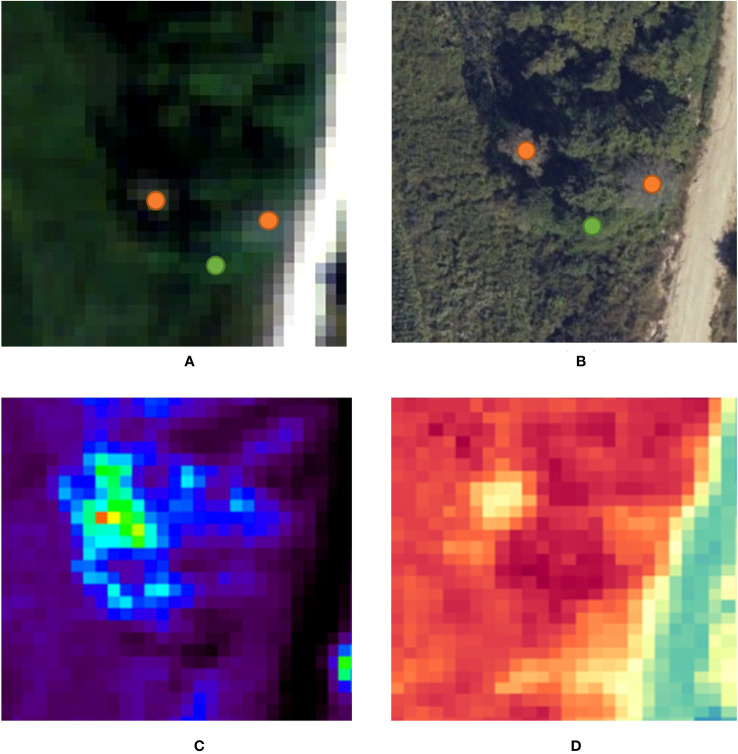
**(A)** Raw hyperspectral data **(B)** Raw CCD data **(C)** NDWI result **(D)** PSRI result.

**Figure 7** shows the high-resolution data and raw hyperspectral data for the experiments on marked trees according to NDWI and PSRI vegetation indices. The experimental results show that the raw hyperspectral data using the vegetation indices cannot distinguish well the differences among the variegated standing trees.

**Figure 8:**
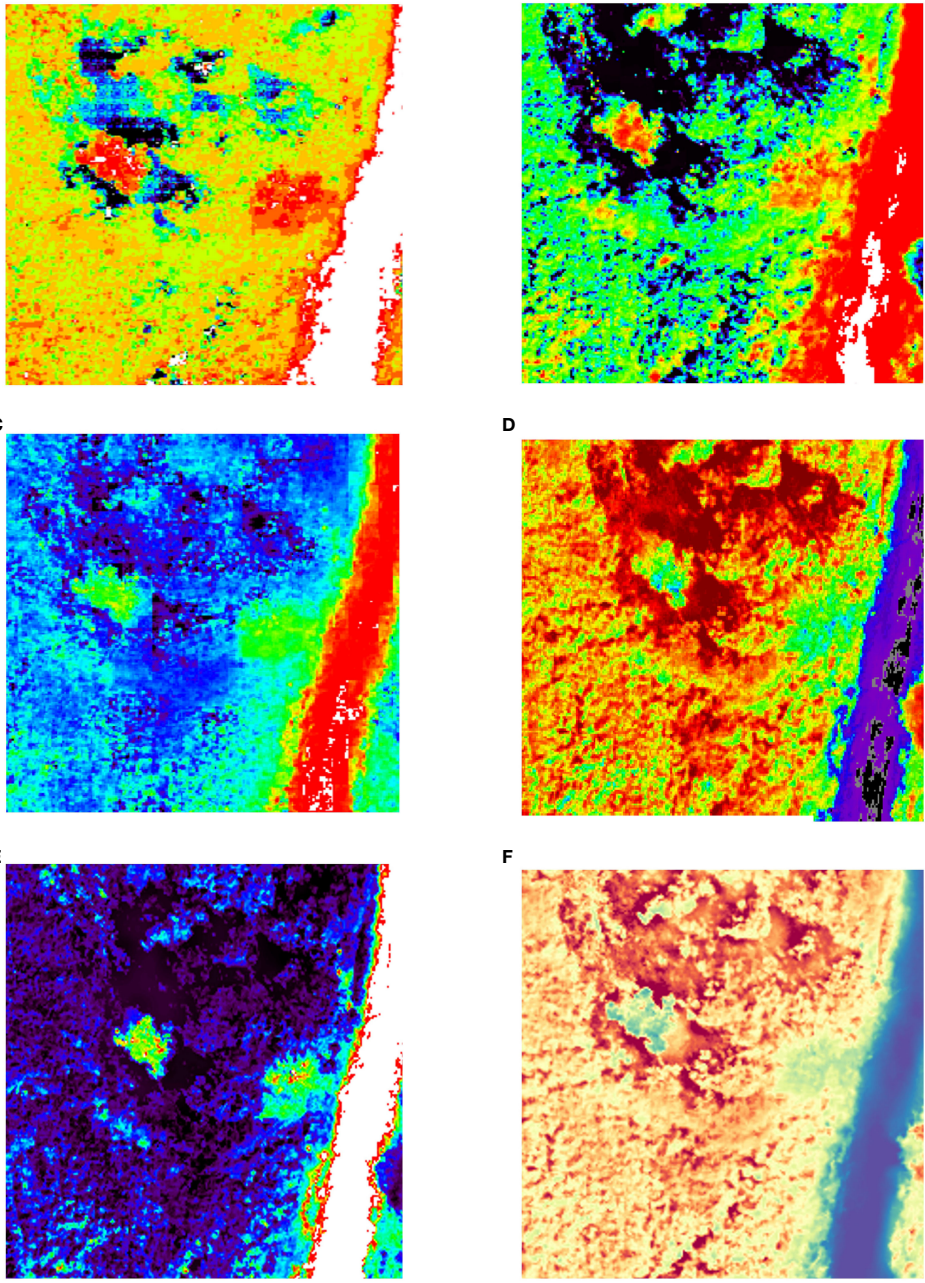
NDWI results. **(A)** HySure result; **(B)** CNMF result **(C)** MAP-SMM result **(D)** PC Spectral Sharpening result. **(E)** GLP result **(F)** Proposed result.

Below **Figure 8** shows the fusion results obtained by choosing the vegetative index NDWI, which is based on the original hyperspectral data, effectively preserving the spectral information and improving the level of spatial detail expression. The MAP-SMM algorithm improves the spatial information capability and effectively preserves the spectral information of the tree canopy as well as the spatial detail expression with good spectral fidelity. The HySure algorithm is able to optimally preserve the shape features of the canopy and can clearly distinguish between discoloured standing trees and healthy trees, but there is a spectral distortion in the parts around the canopy. The CNMF algorithm can portray the general contour shape of the canopy and retain certain spectral information. The PC spectral sharpening algorithm fails to portray well the difference between the discoloured standing trees and the healthy trees, which will have some spectral loss at the edges of their canopy. The GLP results are able to retain the spectral information in the tree canopy and lack some spatial details in the characterization of the canopy profiles. The algorithm presented in this paper characterizes the tree canopy at the level of spectral analysis, clearly distinguishes between standing trees with discoloration and healthy trees and can efficiently preserve spectral components with less spectral distortion.

**Figure 9:**
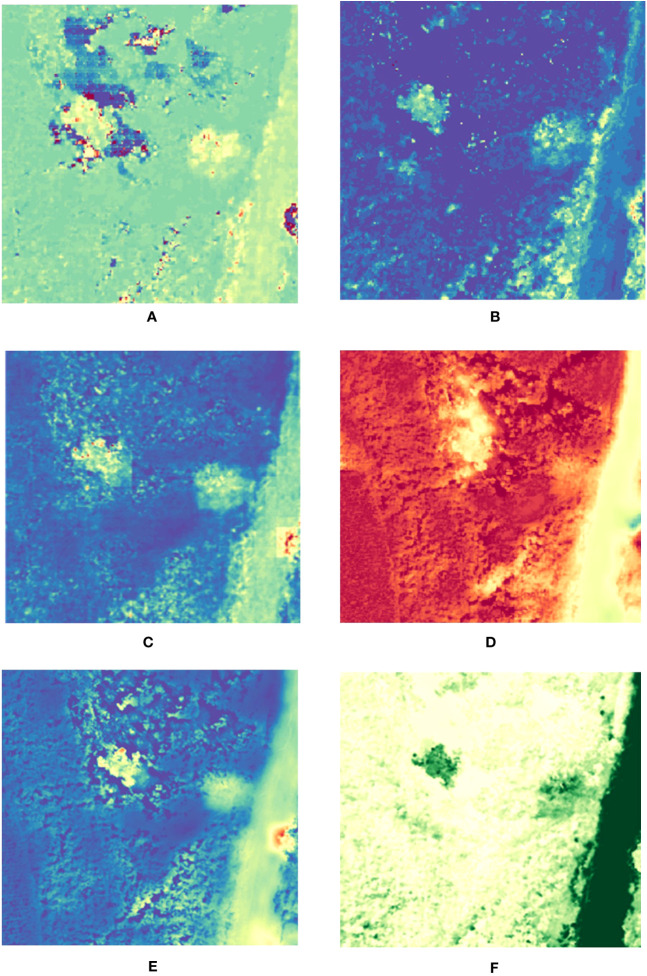
PSRI results. **(A)** HySure result; **(B)** CNMF result **(C)** MAP-SMM result **(D)** PC Spectral Sharpening result. **(E)** GLP result **(F)** Proposed result.

**Figure 9** below shows the fusion results obtained by choosing the vegetation index PSRI. The results of the MAP-SMM algorithm retain the spectral information in the original data and can be combined with the information in the original high-resolution CCD data for a considerably sharper result in the detailed characterization of the canopy level. The fusion results of the HySure algorithm can effectively preserve the spectral information in the tree canopy and can resolve the spectral differences with the tree canopy, improving the spatial detail expression, but there are some spectral distortions around the tree canopy and the shaded parts. The figure shows that the PC spectral sharpening algorithm is less able to preserve the spectral information compared to the other algorithms, while the spatial information is considerably improved. However, it fails to better preserve its spatial expression in the carving of canopy details. The GLP algorithm is able to reflect the approximate details of the tree canopy, and the spectral information is preserved to some extent. The proposed algorithm preserves the spectral information while enhancing the spatial details, and the spectral distortion around the tree canopy is minor.

In summary, the vegetation index can effectively analyse the fusion results from spectral preservation and spatial detail enhancement. The proposed algorithm effectively preserves spectral information while enhancing spatial details and is able to preserve canopy information of variegated standing tree canopies. Among the different algorithms, MAP-SMM works best for canopy detail feature carving, but with some spectral distortion, followed by HySure, CNMF, and GLP algorithms, with PC Spectral Sharpening being the least effective. All six algorithms described above are capable of reconstructing the spatial and spectral information of the canopy of variegated standing trees, which can be distinctly distinguished from other healthy vegetation, based on raw hyperspectral data.

## 4 Discussion

Each year, losses in forest resources and direct or indirect economic losses due to forest diseases and pests are extremely severe. With the rise of remote sensing monitoring technologies, rapid and high-scale monitoring of forest diseases and pests has become possible. CCD images obtained by remote sensing surveillance techniques can clearly show spatial information of decorated trees infected with diseases and pests. However, in order to enable early monitoring of diseases and pests, it is necessary to use hyperspectral images to analyse the changes in their internal chemical properties and thus detect the occurrence of diseases and pests in the early stages of tree infection. With the development of forest pest monitoring technologies, traditional detection methods have struggled to meet the demands of detection resolution and identification accuracy. Therefore, in this paper, we propose an image fusion algorithm based on Airborne data that not only preserves the spectral information of the original image but also effectively improves the spatial resolution of the detection results, which has certain advantages for early detection and recognition of forested trees.

### 4.1 Characteristics of forest pest and disease data

#### 4.1.1 CCD data characteristics

CCD data is a type of image data that can accurately and intuitively show the occurrence and development of forest pests and diseases. Pixel-scale colour analysis can detect anomalous colour changes, such as brown, yellow, reddish-brown, grey, and other colours in CCD image data when trees are affected by pests and diseases. In contrast to hyperspectral techniques, CCD data detection is faster, high-resolution image data acquisition is less difficult, and it is more widely used in forest pest monitoring. How-ever, when significant changes in crown colour are detected, the tree is already in the middle or late stages of pest and disease and can only be managed by cutting and crushing. Therefore, this paper combines hyperspectral data analysis to enable early detection of discoloration in trees.

#### 4.1.2 Spectral data characterization

Hyperspectral data is a class of data that can be multi-band, express spatial and spectral information, and capture the continuum of the target. Hyperspectral data can detect minor changes in the tree spectrum during discoloration, when there are colour abnormalities in the canopy caused by an infestation of trees with pests and diseases. In contrast to CCD data, hyperspectral data can reflect the characteristics of each epoch from the spectral curve, which is widely used in the field of early monitoring of forest pests and diseases. However, the spatial resolution of the hyperspectral images is not as excellent as that of the CCD images, and it is difficult to distinguish tree crowns with similar colours, contours, and additional textures in the raw hyperspectral data. Therefore, this paper combines CCD data analysis to enable early monitoring of tree discoloration.

#### 4.1.3 Fusing algorithm data features

The fused data can preserve the spatial detail texture information of the CCD data, better preserve the coronal edge characterization, and preserve the spectral information of the hyperspectral data.

These fused data can be used not only to analyse the spectral characteristics of the canopy but also to determine the internal stage and extent of the disease. In addition, the fusion results additionally improve the spatial resolution of the images, enabling the detection of anomalous canopies with higher contrast and more pronounced effects, clearer canopy textures and contours more appropriate for the true onset of the disease, and the discrimination of anomalous canopies with similar colours. In this way, the fused data have both strong spatial and extreme spectral resolution. Serve as a valid reference for further research.

### 4.2 Performance analysis of fusion algorithms

Multisource data fusion algorithms are increasingly used in remote sensing monitoring due to technological developments. Fusion analysis of multi-source data can integrate the features and strengths of different types of data and compensate for the shortcomings of individual data. Moreover, the fusion algorithm does not severely increase the computational cost and can considerably increase the detection efficiency and effectiveness. In this paper, we compare the improved fusion algorithm with alternative algorithms and obtain better experimental results.

Therefore, the fusion algorithm combines CCD data and hyperspectral data to detect standing trees with different colors in the experimental region, which preserves the data characteristics of standing trees and improves the resolution of the detection results.

### 4.3 Selection of evaluation indicators

The construction of a fusion quality evaluation system is an important problem in image fusion. At present, most of them are judged by qualitative and quantitative indicators (Pei et al., 2012).

In this paper, airborne remote sensing images are used as the subject of study and the spectral fidelity and spatial resolution between images can be evaluated using selected fusion algorithm metrics. For the application level of forest remote sensing, models for the corresponding evaluation schemes are constructed and designed through the physiological features of different objects. For each evaluation scheme, reasonable weights are assigned according to the requirements of the actual application and the relevant data sources, and finally, a comprehensive evaluation result is obtained. Evaluation metrics can include both application level and comprehensive analysis of the fusion results from a fusion perspective. In this paper, we conduct an analysis and evaluation of experimental results utilizing PSRI and NDWI vegetation metrics to enhance the visual representation of fusion outcomes and assess the applicability of fusion algorithms.

### 4.4 Quantitative and qualitative evaluation

Based on qualitative analysis, it is evident that each algorithm is able to enhance the texture features of the original hyperspectral data to some extent by integrating the results of the canopy detail images. However, the algorithm in this paper excels in de-tailed description and edge preservation.

As can be seen from the error plots of the fusion results, each algorithm has some error with respect to the reference image sampled on the original hyperspectral, and the difference between the proposed algorithm and the reference image is minor. According to the analysis of the quantitative results, the proposed algorithm performs nicely on SAM, CC, and Entropy metrics, and can also perform at a strong level on other quantitative metrics. All fusion algorithms can improve the spectral and spatial resolution of the raw data. The proposed algorithm can effectively distinguish between discolored and normal trees at the spectral level and preserve the canopy details and spectral features of the tree canopy. From the spectral curve level analysis, the fusion algorithm is able to better preserve the trends of the spectral curve and still have certain spectral features within a particular band after fusion compared to the original data. Using a single hyperspectral data for detection, there will be exotic objects with the same spectrum and the same object with a different spectrum, and tree crowns with similar texture profiles will be difficult to distinguish. The use of single CCD data to detect discolored standing trees makes it impossible to determine whether the trees are infested with pests and diseases and at what stage of infection. The final fusion results using a specific vegetation index can extract tree crowns with better results than those obtained from a single data source. Our experimental results show that the fusion algorithm has some adaptability for the detection of standing trees in forest discussions and has promising applications.

### 4.5 Prospects

In this paper, we use a fusion algorithm to fuse CCD data and hyperspectral data to analyze the occurrence and development of forest pests and diseases. Environmental factors such as illumination and topography can have some influence on the acquisition of image information during data acquisition, and the effect of data acquisition directly affects the results computed by the fusion algorithm. Therefore, in the subsequent research, the data collected by the ground base station can be combined to detect and analyze the tree crown characteristics by multi-source data fusion so that the abnormal spectrum of the collected crown can be more accurate, the abnormal spectral characteristics can be extracted more real and reliable, and the application ability of the fusion algorithm in the aspects of pest and disease and forest control can be im-proved. In addition, there is a lack of a well-defined vegetation index suitable for assessing the extent of early pest and disease damage. An appropriate vegetation index can be designed based on the spectral wavelength of a particular object, and the fusion result can be effectively distinguished from the spectral fidelity, which can be used as an essential evaluation metric for the fusion result of forest remote sensing. At the same time, the results of the vegetation index are also affected by light and other relevant factors, and the influence of environmental factors should be considered in the future and differentiated according to the actual situation.

## 5 Conclusions

It has been shown that the proposed fusion algorithm can be used to perform fusion experiments using onboard remote sensing data in the experimental sample area, and the fusion results verify the effectiveness of the proposed method, which has some application value. Comparison and evaluation of the proposed algorithm with five additional fusion algorithms through various evaluation metrics show that the pro-posed algorithm introduces guided filtering based on multi-resolution analysis and improves the spatial detail features while preserving the canopy spectrum. The fusion results in a large spatial resolution as well as a large spectral resolution. The research results can provide good technical support for the practical application of forest remote sensing data fusion, and lay the foundation for promoting the scientific, automatic and intelligent forestry control.

The limitation of this study lies in the lack of comprehensive analysis of experimental results using multi-source data. In the future, effective integration of advantages from all parties, improved efficiency in data utilization, and promotion of result analysis can be achieved through cross-scale fusion of air and earth observation data. Additionally, the qualitative evaluation method adopted in this study has certain limitations that moderately affect result accuracy. Therefore, it is necessary to consider extracting abnormal spectral characteristics and designing corresponding vegetation indices and evaluation criteria for different tree species and infection stages to achieve varying levels of detection. This will help enhance monitoring capabilities and address practical application challenges.

## Data availability statement

The raw data supporting the conclusions of this article will be made available by the authors, without undue reservation.

## Author contributions

HZ: Conceptualization, Funding acquisition, Methodology, Project administration, Resources, Writing – original draft. YW: Conceptualization, Formal Analysis, Methodology, Visualization, Writing – original draft. WW: Investigation, Writing – review & editing. JYS: Methodology, Supervision, Validation, Writing – review & editing. GL: Formal Analysis, Writing – original draft. JS: Data curation, Writing – review & editing. HS: Data curation, Writing – review & editing.
